# Make Your Cake and Eat It: Refueling of Immune Fitness in AML Post Allo-HCT Using Baking Soda

**DOI:** 10.20900/immunometab20210005

**Published:** 2021-01-21

**Authors:** Alex Tonks

**Affiliations:** Department of Haematology, Division of Cancer & Genetics, School of Medicine, Cardiff University, Wales CF14 4XN, UK

**Keywords:** AML, GvL, immunomodulation, metabolism, T cell, bicarbonate of soda

## Abstract

Although there has been a recent renaissance in the availability of new therapeutic options for patients with acute myeloid leukemia (AML), survival rates remain low coupled with a high incidence of relapse. Enhancing T cell and immune function has become an effective therapeutic approach in hematological malignancies. However, AML cells can modulate the bone marrow microenvironment by changing extracellular nutrient and biochemical availability which can metabolically regulate immune function. Here we review the findings by Uhl et al. showing that T cell metabolism and function can be boosted by treatment with sodium bicarbonate to counteract the metabolic changes induced by lactic acid produced by leukemia cells.

## Introduction

Acute myeloid leukemia (AML) is an aggressive hematological malignancy arising from developmental arrest of cells of the myeloid lineage. The disease is characterized by a dominant clone of immature cells which rapidly accumulates in the bone marrow (BM) and peripheral blood resulting in the failure of normal hematopoiesis. The disorder is highly heterogeneous with distinct morphological, cytogenetic, and genetic abnormalities making the disease difficult to treat [1,2]. Clinical outcomes are generally poor, averaging around 45–50% survival at five years in younger patients (under 60 years) and 15–20% for those over 60 years if treated with curable intent [3]. Clinical outcomes for AML patients have improved particularly for those individuals under 60 years. This is largely due to better supportive care, but an improved understanding of relapse risk, greater availability of allogeneic hematopoietic cell transplantation (allo-HCT) and approval of several targeted therapies (particularly over the last 3 years) have also contributed [4]. However, up to 50% of AML patients relapse following allo-HCT depending on disease status and characteristics [5] and prognosis remains dismal at below 20% [6]. Thus, long term remission in AML is generally not durable and there is an urgent need to establish new treatments to prevent relapse. One approach is to improve the graft-versus-leukemia (GVL) effect.

The recent study by Uhl et al. published in Science Translational Medicine has suggested that the failure of T cells in the allo-HCT recipient maybe related to impaired activation and metabolic activity of T cells imparted indirectly by the leukemia cells themselves [7]. To examine the links between T cell function, metabolic activity and AML relapse, the authors isolated T cells from AML patients who relapsed after allo-HCT and assessed metabolic fitness of CD8^+^ T cells. A profound reduction in glycolytic activity and oxidative phosphorylation was observed. Interestingly, using a murine model, culture of supernatant from leukemia cells with murine T cells was able to recapitulate reduction in metabolic fitness which was accompanied by reduced anti-tumor activity and suggest that a soluble factor was responsible. The concept that the metabolic state of the microenvironment is regulated by the activity of the leukemia (or cancer) cell is nothing new. Altering the availability of nutrients or chemicals within the microenvironment creates metabolic competition between immune cells, mesenchymal stromal cells (MSC) and cancer cells [8], with each cell utilizing the nutrients for different means (e.g., proliferation, DNA repair). For example, we recently showed that reactive oxygen species (ROS) which is inappropriately produced by NAPDH oxidase (NOX2) on the surface of AML cells, can alter AML cell metabolism to support proliferation [9]. In-deed, ROS can also affect anti-tumor responses, acting as second messengers within T cells, controlling cell proliferation and clonal expansion ([10] and reviewed in [11,12]). Given the plethora of nutrients and chemicals produced by AML cells, it was therefore not surprising that changes in metabolites of AML cell and T cell culture supernatants were observed when analyzed by mass spectrometry. Uhl et al. noticed lactic acid levels to be highly abundant in the supernatant of leukemia cells in vitro when compared to control medium or supernatant derived from a T cell culture. The plasma of patients at primary AML diagnosis had lower levels of lactic acid and correlating CD8^+^ T cells exhibited a robust metabolic and cytokine profile compared to patients with AML relapse. Unfortunately, it was not tested what the levels of lactic acid were in de novo AML diagnostic samples compared to normal HSC or indeed the function of CD8^+^ T cells vs T cells from healthy individuals. Lactate levels have previously been shown to be increased in AML blasts at diagnosis [9] and acute leukemias [13]. Regardless, Uhl et al. showed significant increases in lactic acid in patients with AML relapse after allo-HCT but not in patients with AML who entered remission following allo-HCT. The observed increases in lactic acid level were associated with acidification of the medium, lower pH and impaired T cell proliferative capacity and anti-tumor immunity. It was pleasing to see that that T cell proliferation was not affected by extracellular acidification when using hydrochloric acid, suggesting that acidification of the supernatant alone was not responsible for the observed effects of lactic acid. It was already known that high levels of lactic acid have an inhibitory effect on human T cell effector function [14] but it remains unclear whether the negative impact of lactic acid secreted by AML cells on T cell function is only found in the context of AML relapse following allo HCT, and if so why is this the case?

To provide pre-clinical evidence of the efficacy of antagonizing metabolic acidosis in AML and the lactic acid induced effects on T cells, Uhl et al. aimed to assess T cell responses and leukemia development in vivo. To achieve this, the authors used a murine model coupled with sodium bicarbonate (NaBi; aka NaHCO_3_, baking soda or bicaNorm) treatment. Here AML bearing (WEHI-3B)-mice that underwent allo-HCT were administered NaBi. As expected NaBi did not have any direct toxic effects on AML cells in vivo. However, in the presence of T cells, the animals survived longer and had improved metabolic fitness and T cell function though syngeneic murine transplantation control experiments were not included here (and elsewhere in the study). Uhl et al. acknowledge the limitations of the murine model used in this study, though confirmatory studies were reproduced in the genetic FLT3-ITS/MLL-PTD based GvL model. However, the study finishes with tantalizing data building on the above preclinical studies. Ten patients suffering from relapse following allo-HCT and received donor lymphocyte infusions were treated with oral NaBi (BicaNorm is clinically used to antagonize metabolic acidosis in patients) and improvements in metabolic and immune profiles of isolated T cells was observed. Whilst this approach seems promising, further discussion on the effects of BicaNorm treatment between donor T cells and autologous patient T cells were required. As stated above, high levels of lactic acid have an inhibitory effect on human T cell effector function [14], so it is not clear why Uhl et al. thought that lactic acid would negatively impact allogeneic donor T cells but not autologous patient T cells. Further, we are not confident on the clinical impact of NaBi treated patients in such a small study and as one would expect, the authors suggest that improved and more extensive clinical studies will need to be performed. It would be prudent to include in the trial patients who had not received allo-HCT and relapsed with AML. Further, given the heterogeneity in AML and subtypes that have metabolic alterations (e.g., IDH mutations), such studies will need to be on large scale to ensure appropriate power, especially considering that the defined group would be relapsing following allo-HCT.

Whilst the study shows some correlation of lactic acid levels with CD8^+^ T cell function/activity, the BM microenvironment is a complex tissue; in addition to tumor and the extracellular matrix, the HSC niche may comprise different cell populations such as MSC, endothelial cells, osteoblasts as well as progeny of HSCs and other immune cell subtypes (NK cells, CD4^+^ T cells, regulatory T cells, γδT and iNKT cells etc.). It remains to be understood what the role of increased lactic acid levels are in this complex environment regarding re-invigorating immune function and affects AML blast survival. Further, mechanistically, whilst the authors show reduced glycolytic activity in AML correlates with lactate release, further insight into this mechanism may provide additional therapeutic targets. For example, it would be interesting to determine whether targeting the pathways that lead to increased lactate levels is also a viable alternative in AML patients following allo-HCT and relapse. Monocarboxylate transporters (MCT) are responsible for transmembrane lactate trafficking, with MCT1 involved in both import and export, MCT2 import and MCT4 primarily export of lactate, with all these isoforms reported to be overexpressed in different tumors (reviewed in [15]). Here, inhibiting T cell uptake of lactic acid using MCT1 inhibitor led to normalization of glycolytic activity comparable to untreated T cells. Indeed, many of the effects of lactate on T cell immune function can be recapitulated through modulating MCT activity and again provide alternative strategies to antagonize the acidosis in the patient.

**Figure 1:**
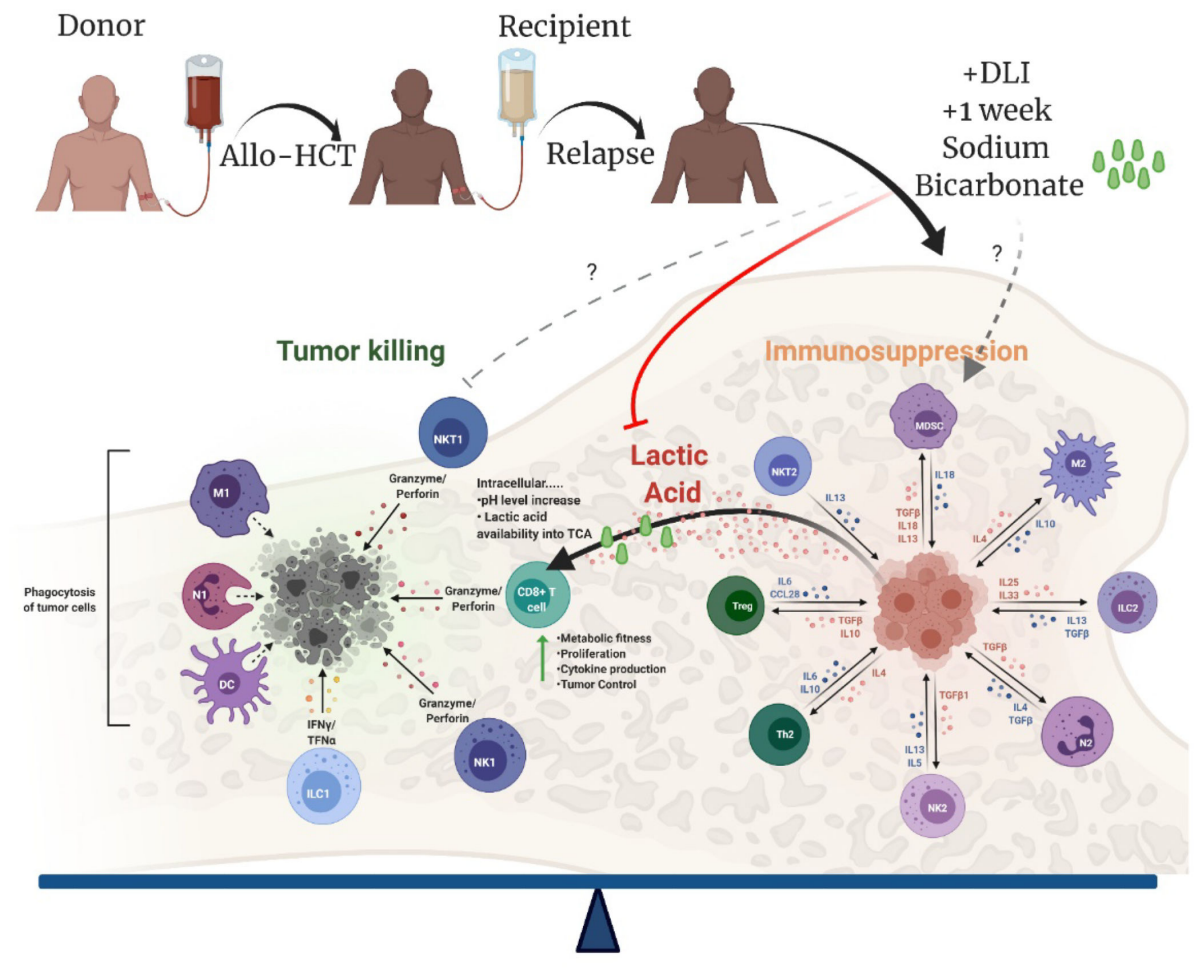
Potential effects of sodium bicarbonate treatment on immune cells in an AML lactic acid producing environment following an allogeneic hematopoietic cell transplant (HCT). The mechanisms for T cell metabolic boost are based on findings from Uhl et al. [7]. Following an allo-HCT, elevated lactic acid levels produced by AML blasts in the BM microenvironment decreases T cell intracellular pH and impairs metabolic fitness of T cells; effects are restored by sodium bicarbonate treatment leading to improved T cell fitness and function and further metabolism of additional lactic acid into the tricarboxylic acid cycle (TCA). Uhl et al. focused on CD8^+^ T cell function, but effects of sodium bicarbonate treatment on other immune subtypes and BM microenvironment are not currently known.

In conclusion, the study of Uhl et al. highlights the importance of relapse in AML following allo-HCT. They present compelling data that provide a pharmacological strategy to enhance GvL effects in patients relapsing following allo-HCT. The data shows that treatment using sodium bicarbonate overcomes metabolic reprogramming of transferred T cells to improve tumor control (Figure 1). It is interesting to speculate that in the right context, treatment with sodium bicarbonate or agents that improve immune metabolic fitness may augment current therapies such as CAR-T [16], checkpoint inhibition [17], or blockade of immunosuppressive “don’t eat me signals” [18] and ultimately further improve AML patient outcome.

## Abbreviations

AML: acute myeloid leukemia; allogeneic hematopoietic cell transplantation (allo-HCT)
BM: bone marrow
GvL: graft-versus-leukemia
HSC: hematopoietic stem cell
MCT: monocarboxylate transporters
MSC: mesenchymal stromal cells
ROS: reactive oxygen species
